# Crowd-Aware Mobile Robot Navigation Based on Improved Decentralized Structured RNN via Deep Reinforcement Learning

**DOI:** 10.3390/s23041810

**Published:** 2023-02-06

**Authors:** Yulin Zhang, Zhengyong Feng

**Affiliations:** School of Electronic Information Engineering, China West Normal University, Nanchong 637009, China

**Keywords:** robot navigation, deep reinforcement learning, RNN, spatio-temporal graphs, coarse-grained local maps

## Abstract

Efficient navigation in a socially compliant manner is an important and challenging task for robots working in dynamic dense crowd environments. With the development of artificial intelligence, deep reinforcement learning techniques have been widely used in the robot navigation. Previous model-free reinforcement learning methods only considered the interactions between robot and humans, not the interactions between humans and humans. To improve this, we propose a decentralized structured RNN network with coarse-grained local maps (LM-SRNN). It is capable of modeling not only Robot–Human interactions through spatio-temporal graphs, but also Human–Human interactions through coarse-grained local maps. Our model captures current crowd interactions and also records past interactions, which enables robots to plan safer paths. Experimental results show that our model is able to navigate efficiently in dense crowd environments, outperforming state-of-the-art methods.

## 1. Introduction

With the development of artificial intelligence, mobile robots can be seen everywhere in daily life, and how to efficiently navigate in the unstructured environment of human life will become more and more important. Traditional methods generally only consider static obstacles, which often lead to unsafe and unnatural behaviors in dynamic crowd environments [1,2]. To solve this problem, we should make robots obey the cooperation rules of humans [3]. For example, the robot should move smoothly and not cut through the crowd.

Navigation with social etiquette is a challenging task. Since there is generally no explicit communication between the robot and other agents, it is necessary for the robot to understand and predict the behavior of other agents. However, there are both moving agents and temporarily static agents in a crowded environment. There are various explicit and implicit interactions among agents, which are usually difficult to model [4].

There are two main types of methods to solve this problem in early research. The first type is the model-based method, which selects the best action through one-step interaction rules, such as optimal reciprocal collision avoidance (ORCA) [5] and social force model (SFM) [6]. The second type is based on prediction methods, by predicting the future trajectories of other agents, and then planning a best path for the robot, such as [7,8]. However, in dynamic dense scenes, these methods will cause the problem of robot freezing. Usually, feasible paths still exist in this scenario [9,10], but the difficulty of planning will be greatly increased.

In recent years, with the rapid development of machine learning, deep reinforcement learning methods have been widely used in crowd navigation [11,12,13,14,15,16]. Since the deep reinforcement learning strategy can implicitly encode the interaction and cooperation among agents, the navigation performance has been greatly improved. Although recent studies have made significant progress, these strategies still have two shortcomings: (1) Initialized by ORCA through imitation learning, the strategy inherits the drawbacks of ORCA [13,14]; (2) Only the interactions between robot and humans is considered, and the interactions between humans and humans is not considered [17]; (3) Only low-density dynamic environments are considered, and the performance will drop in high-density dynamic environments [13,14,17]. Due to these deficiencies, these methods become less effective in more challenging settings.

In this work, we propose to build more comprehensive interactions rather than just first-order Robot–Human interactions. We model Robot–Human interactions through spatio-temporal graphs, and Human–Human interactions through local coarse-grained maps. These interactions are processed through an attention mechanism to learn the relative importance of surrounding humans, as well as the relative importance of Human–Human interactions. Our method is trained via model-free reinforcement learning, does not require imitation learning with an expert policy, and thus does not converge to a locally optimal policy. We conduct extensive experiments in dynamic dense crowd environments, and the experimental results show that our model performs better than previous methods in terms of navigation success rate and time efficiency.

## 2. Related Works

### 2.1. Model-Based Methods

Early works have accomplished navigation in dynamic environments by designing specific interaction models. One is a reaction-based method, such as RVO [18] and ORCA [5]. This type of method is based on the speed obstacle area method [19], and can obtain the joint collision avoidance speed under the assumption of reciprocity. The other is the social force model [6], which simulates the interactions among agents through goal driving force, repulsive force, and attractive force. These model-based methods rely heavily on hand-crafted functions, which may require different parameters in different scenarios, and finding a suitable parameter for dynamic-dense scenes is difficult [20].

### 2.2. Learning-Based Methods

With the rapid development of machine learning, learning-based methods have been extensively studied, which combine supervised learning and reinforcement learning, and have shown promising results in crowd navigation [11,12,13,14]. These works use simulated environments to collect robot navigation experiences, and then use these experiences to update value policies. These strategies are given the state transition probabilities of all agents, then calculate the value of all possible next states, and finally choose the action that maximizes the value. When training the value network, imitation learning is first performed, that is, the trajectory generated by ORCA is used to initialize the network parameters. The network parameters are then tuned using reinforcement learning. These methods must know the state transition probabilities of all humans, but state transitions of humans are usually difficult to model. Furthermore, since such methods employ imitation learning for initialization, they often inherit the shortcomings of the demonstration strategy.

### 2.3. Spatio-Temporal Graph Methods

In recent years, some works have represented the problem into components and their spatio-temporal interactions through spatio-temporal graphs, which have achieved good results in fields such as trajectory prediction and human tracking [21,22,23]. Jain et al. [24] converted arbitrary spatio-temporal graphs into RNN networks, proposing a method called structured RNN. Liu et al. [17] proposed a decentralized RNN network, which simulates the spatio-temporal interactions between the robot and other agents through the nodes and edges of the spatio-temporal graph, and achieved good results in crowd navigation. However, this method only focuses on the interactions between the robot and other agents, ignoring the interactions among other agents. Based on these models, we design a new neural network that considers not only Robot–Human interactions, but also Human–Human interactions.

## 3. Approach

In this section, we first introduce how to represent the problem of crowd navigation with deep reinforcement learning. Then, the spatio-temporal graph is used to model the Robot–Human interaction, and the local coarse-grained map is used to model the Human–Human interaction. Finally, our LM-SRNN neural network structure is derived according to the previous interactive method.

### 3.1. Problem Formulation

In this work, we view crowd navigation as a partial Markov decision process. $s_{t}$ and $a_{t}$ represent the state and action of the agent (robot or human) at time *t*, respectively. The state representation of the agent is similar to [13,25], each agent has an observable state that can be observed by other agents, including position ${(p}_{x},p_{y})$, velocity ${(v}_{x},v_{y})$, and radius r; each agent also has a hidden state that can only be observed by itself, including the target position $\left(g_{x},g_{y}\right)$, maximum speed $v_{max}$, and heading angle θ. Our task is to deal with the problem of a robot navigating in a scene with n people. The input state of the robot is defined as $S_{t}=\{s_{0}^{t},s_{1}^{t},\;\ldots \;,s_{n}^{t}\}$, $s_{0}^{t}$ is the entire state of the robot, $s_{i}^{t}\;(i=1,2,\;\ldots \;,n)$ is the observable human state. The action of the agent consists of the speed of the x-axis and the speed of the y-axis, $a_{t}=(v_{x},v_{y})$.

In each episode, the robot starts from the initial state $S_{0}$. At time t, the robot chooses an action $a_{t}$ according to the policy $\pi (a_{t}|S_{t})$, and then moves to the next state $S_{t+1}$. In return, the robot gets a reward $r_{t}$. At the same time, humans also choose their own actions according to their own strategies and move to the next state. The whole process may last the maximum length of an episode, or it may end early due to a collision or reaching the goal.

We follow the reward function formulation in [17], rewarding the robot for task achievement while penalizing the robot for collisions with humans or getting too close to humans. Additionally, in order to guide the robot, the behavior of approaching the goal is rewarded:

(1) $$\left\{ \begin{array}{l} {-20,\;\;\;\;\;\;\;\;\;\;\;\;\;\;\;\;\;\;\;\;\;\;\mathrm{if}\;d}_{\min}^{t}<0\\ 2.5(d_{\min}^{t}-0.25),{\;\;\;\mathrm{if}\;0\;<\;d}_{\min}^{t}\;<\;0.25\\ {10,\;\;\;\;\;\;\;\;\;\;\;\;\;\;\;\;\;\;\;\;\;\;\;\;\;\;\mathrm{if}\;d}_{goal}^{t}{\;<\;r}_{robot}\;\\ {2(d}_{goal}^{t-1}{-d}_{goal}^{t}),\;\;\;\;\;\;\;\mathrm{otherwise}\; \end{array}\right.,$$

where $d_{\min}^{t}$ is the minimum distance between the robot and the human at time t, and $d_{goal}^{t}$ is the Euclidean distance between the robot position and the target position at time t. The total return at time t is defined as:

(2) $$R_{t}=\sum_{k=0}^{\infty }\gamma^{\left(k\right)}r_{t+k},$$

where γ∈(0,1) is the discount factor. The value function is defined as $V(S_{t})=E(R_{t}|S_{t}=S)$.

### 3.2. Modeling of Robot–Human Interactions 

**Figure 1:**
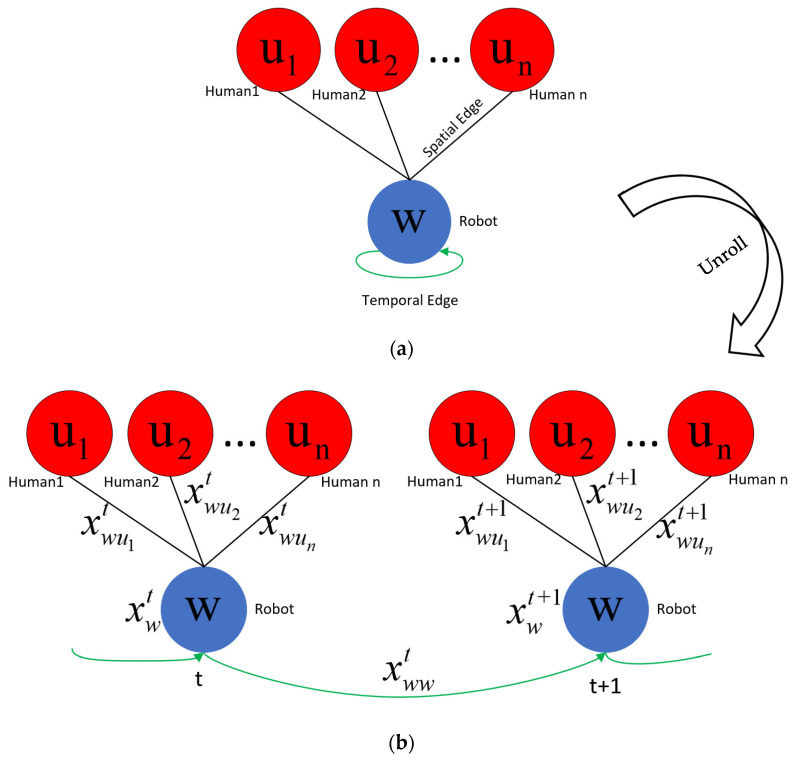

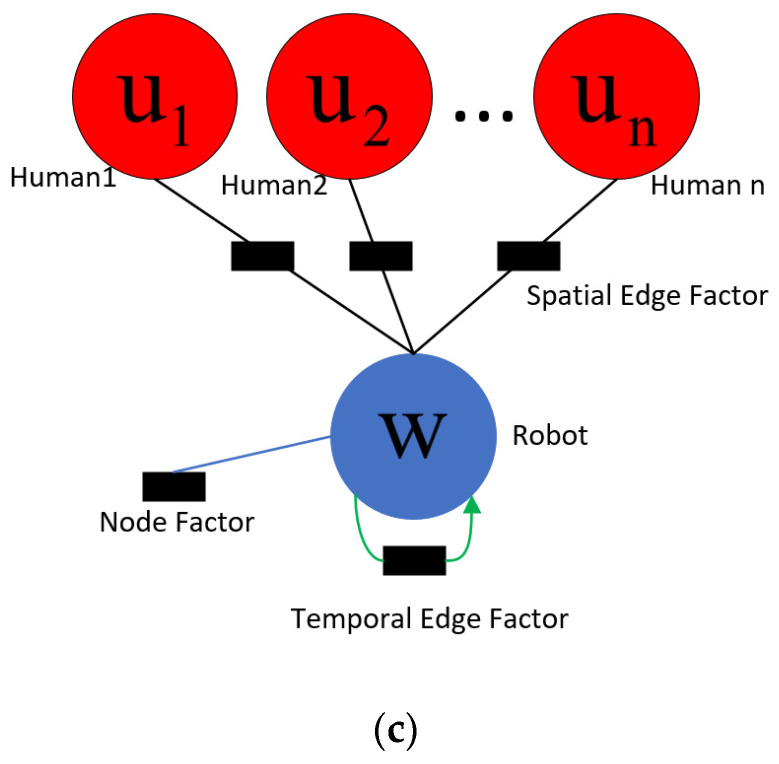
Spatio-temporal graph of robot interaction with humans during navigation. (**a**) A spatio-temporal graph capturing Robot–Human interactions. We use w to denote the robot node, and $u_{i}$ to denote the i-th human node. (**b**) The spatio-temporal graph unfolded in two steps. At time step t, the spatial interaction feature between the robot and the *i*-th human is $x_{wu_{i}}^{t}$. The time feature of the robot is $x_{ww}^{t}$. The robot node feature is $x_{w}^{t}$. (**c**) The factor graph corresponding to the spatio-temporal graph.

We model the spatio-temporal interactions between robot and humans through a decentralized spatio-temporal graph. Our spatio-temporal graph is expressed as $G=(V,{ℰ}_{S},{ℰ}_{T})$, where 𝒱 represents the node of the agent’s own state information, ${ℰ}_{S}$ represents the spatial relationship edges of different agents at the same time step, and ${ℰ}_{T}$ represents the temporal relationship edges of the same agent at adjacent times step. Figure 1a shows an example spatio-temporal graph of a robot interacting with n humans in a complex scene. Since each individual’s behavior is only influenced by nearby humans, rather than all humans, we prune the spatiotemporal edges between humans and model them in a more concise and effective way in Section 3.3. Figure 1b shows the same spatio-temporal graph unfolded in two steps. In the unfolded spatio-temporal graph, robot nodes at a given time t are connected with other human nodes to form undirected spatial edges; robot nodes at adjacent time steps are connected to form undirected temporal edges.

In the interactions between robot and humans, node features can represent the state information of robot and humans, such as position, velocity, radius, target position, etc. Edge features represent their relative direction and distance. The state of a node is affected by its own interactions with other nodes, forming a complex system. The interactions are usually parameterized by factor graphs, which can decompose complex problems in spatio-temporal graphs into simpler ones. As shown in Figure 1c, the one-way interactive robot navigation strategy is expressed as robot node factors, spatial edge factors, and temporal edge factors through a factor graph. The black rectangles in the figure represent factors with parameters that need to be learned.

More specifically, the $x_{w}^{t}$ is the robot state $\left[p_{x}{,p}_{y}{,v}_{x}{,v}_{y}{,r,g}_{x}{,g}_{y}{,v}_{\max},\theta \right]$, the feature $x_{\mathrm{ww}}^{t}$ is the robot speed ${(v}_{x}{,v}_{y})$, and the feature $x_{{\mathrm{wu}}_{i}}^{t}$ is represented by the relative positional relationship vector between the robot and the human $\left(p_{x}^{i}-p_{x},p_{y}^{i}-p_{y}\right)$. Since all Robot–Human spatial edges are semantically similar, we let them share a factor, thereby reducing parameters.

### 3.3. Modeling of Human–Human Interactions 

Everyone will have an impact on the robot’s decision-making, but human behavior will be affected by other people. Therefore, it is extremely important to model the interaction among humans. If all the interactions among human are modeled in detail, the time complexity and space complexity of the algorithm will be very high, which is difficult to run in real-time in large-scale and complex scenes [26]. Inspired by study [14], we model interactions among humans through a local coarse-grained map.

**Figure 2:**
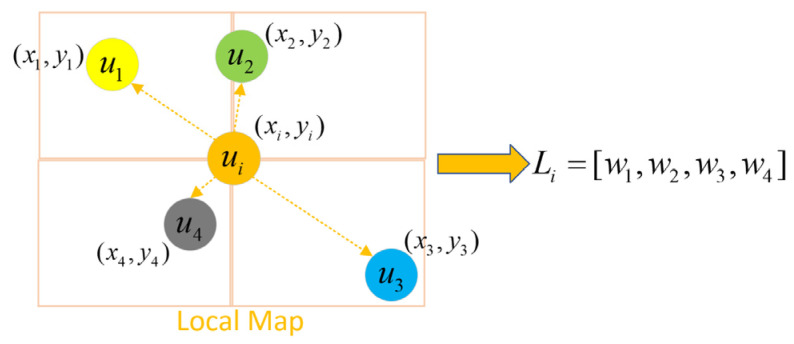
Local map representation of human i being influenced by other humans. where $u_{i}$ represents a human, and $\left(x_{i},y_{i}\right)$ represents the position of human i.

As shown in Figure 2, for each human, we only consider other humans in the neighborhood of size N, and build an N * N * 3 local map $L_{i}$ centered on each human i to encode the presence and speed of neighbors:

(3) $$\begin{array}{l} L_{i}(a,b)=\sum_{j\in N_{i}}\delta_{AB}[x_{j}-x_{i},y_{j}-y_{i}][v_{xj},v_{yj},1]\\ \;\;\;\;\;\;\;\;\;\;\;\;\;=\left\{ \begin{array}{l} \sum_{j\in N_{i}}[v_{xj},v_{yj},1],\;\;\;if-A<x_{j}-x_{i}<A\;and-B<y_{j}-y_{i}<B,\\ -\infty ,\;\;\;\;\;\;\;\;\;\;\;\;\;\;\;\;\;\;\;\;otherwise \end{array}\right. \end{array}$$

where $\left[v_{xj},v_{yj},1\right]$ is the local state vector of human j, $\delta_{AB}[x_{j}-x_{i},y_{j}-y_{i}]$ is an indicator function, only when the relative position (∆x, ∆y) is in the cell (A, B), the indicator function is equal to 1, and $N_{i}$ is a group of adjacent humans around the i-th human.

### 3.4. Neural Network

**Figure 3:**
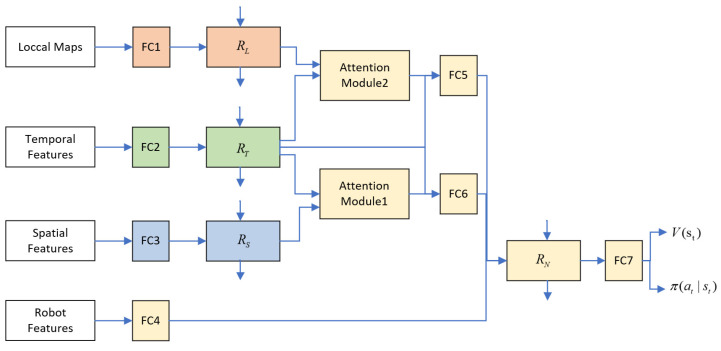
LM-SRNN neural network structure. FC* represents the fully connected layer.

According to study [24], the RNN network structure of Robot–Human interactions can be derived through the factor graph of the spatio-temporal graph. We process the interactions among human through an RNN network, so as to remember the previous interaction information. Our overall neural network structure is shown in Figure 3, $R_{N}$ represents the robot node, $R_{S}$ represents the spatial edge between the robot and the human, $R_{T}$ represents the temporal edge of the robot trajectory change, and $R_{L}$ is the node of Human–Human interactions around the robot.

Before the spatial features are provided to the spatial interaction RNN $R_{S}$, they will first be processed nonlinearly through a fully connected layer (FC3) to obtain the Robot–Human spatial interaction features at the current moment:

(4) $$ES^{t}=\psi_{S}(X_{\mathrm{wu}}^{t};W_{S}),$$

where $\psi_{S}(.)$ is a fully connected layer with ReLU activation and $W_{S}$ is the network weight. $X_{\mathrm{wu}}^{t}$ is the feature of spatial interaction between the robot and all other humans at time t, $X_{\mathrm{wu}}^{t}=[x_{{\mathrm{wu}}_{1}}^{t},\;x_{{\mathrm{wu}}_{2}}^{t},\;\ldots \;,\;x_{{\mathrm{wu}}_{n}}^{t}{]}^{T}$, where n is the total number of humans. Then the embedding vector $ES^{t}$ is provided to the RNN unit to obtain the spatial interaction information with memory:

(5) $$H_{wu}^{t}=RNN_{S}(H_{wu}^{t-1},ES^{t}),$$

where $H_{wu}^{t}{=[h}_{{\mathrm{wu}}_{1}}^{t}{,\;h}_{{\mathrm{wu}}_{2}}^{t},\;\ldots {\;,\;h}_{{\mathrm{wu}}_{n}}^{t}]$ is the hidden state of the RNN at time t for the spatial interaction between the robot and all humans.

Similar to $R_{S}$, the temporal features will also be processed nonlinearly (FC2) before being provided to the temporal interaction RNN $R_{T}$, and then processed by the RNN unit to obtain the robot’s own trajectory variation:

(6) $$h_{ww}^{t}=RNN_{T}(h_{ww}^{t-1},ET^{t}),\;\;\;ET^{t}=\psi_{T}(x_{ww}^{t};W_{T}),$$

where $h_{ww}^{t}$ is the hidden state of temporal interaction RNN at time t.

Compared with $R_{S}$ and $R_{T}$, the Human–Human interaction RNN $R_{L}$ contains information of all Human–Human interactions, which is very informative. First, we input the local coarse-grained map in Section 3.3 into a fully-connected layer (FC1), and then provide the results to RNN units to obtain Human–Human interaction information with memory:

(7) $$H_{L}^{t}=RNN_{L}(H_{L}^{t-1},LM^{t}),{\;\mathrm{LM}}^{t}=\psi_{L}(L^{t};W_{L}),$$

where $L^{t}={\left[L_{1}^{t},L_{2}^{t},\;\ldots \;,L_{n}^{t}\right]}^{T}$ is the local coarse-grained map of all humans at time t, ${\;\mathrm{LM}}^{t}$ is the interaction data after nonlinear processing, and $H_{L}^{t}$ is the hidden state of the RNN of Human–Human interaction features at time t.

To learn the relative importance of each human, we feed the output of the spatial interaction RNN and the temporal interaction RNN into an attention module. This module assigns attention weights to each spatial edge:

(8) $$\alpha_{S}^{t}=soft\max(\frac{n}{\sqrt{d}}H_{wu}^{t}W_{1}{\left(h_{ww}^{t}W_{2}\right)}^{T}),$$

where d is the hyperparameter of attention size and $W_{1}$ and $W_{2}$ are the weight matrices of the linear transformation. The attention mechanism here is similar to the one in [27]. This attention module outputs a weighted sum of spatial edges:

(9) $$HS_{attention}^{t}={\left(H_{wu}^{t}\right)}^{T}\alpha_{S}^{t},$$

To learn the relative importance of interactions among surrounding humans, we feed the output of the Human–Human interaction RNN and the temporal interaction RNN into another attention module. This module assigns attentional weights to each set of interaction features:

(10) $$HL_{attention}^{t}={\left(H_{L}^{t}\right)}^{T}\alpha_{L}^{t},$$

The role of the robot node RNN $R_{N}$ is to process all the previous information and determine the action and value of the robot at time t. First, $HS_{attention}^{t}$ and $h_{ww}^{t}$ are embedded and connected (FC6), and the same processing is performed on $HL_{attention}^{t}$ and $h_{ww}^{t}$(FC5):

(11) $$TS^{t}=\Phi_{S}(HS_{attention}^{t},h_{ww}^{t}),{\;\mathrm{TL}}^{t}=\Phi_{L}(HL_{attention}^{t},h_{ww}^{t}),$$

where $\Phi_{S}(.)$ and $\Phi_{L}(.)$ are the embedding function with ReLU activation. The result of the embedding is then connected to the robot node state, and the connection result is input to $R_{N}$:

(12) $$h_{w}^{t}=RNN_{N}(h_{w}^{t-1},[N^{t},TS^{t},TL^{t}]),\;\;\;N^{t}=\psi_{N}(x_{w}^{t};W_{N}),$$

where $\psi_{N}(.)$ is a fully connected layer with ReLU activation (FC4), and $N^{t}$ is the result of a nonlinear transformation of the robot state.

Finally, $h_{w}^{t}$ is fed into a fully connected layer (FC7) to obtain the value $V(S_{t})$ and the policy $\pi (a_{t}|S_{t})$. We train the entire model using the proximal policy optimization (PPO) algorithm proposed in [28], and the policy and value functions are continuously updated during the training process.

## 4. Experiments

### 4.1. Implementation Details

The local map of Human–Human interactions is a 4 × 4 grid centered on each human, and each cell has a side length of 1 m. The output dimensions of $\psi_{L}$, $\psi_{S}$, and $\psi_{T}$ are (5, 64), (1, 64), and (5, 64), respectively. The dimensions of the hidden units of $R_{L}$, $R_{S}$, and $R_{T}$ are (5, 256), (1, 256), and (5, 256). The output dimensions of $\Phi_{S}$, $\Phi_{L}$, and $\psi_{N}$ are all (1, 64). The dimension of the final $R_{N}$ hidden unit is (1, 192). Our strategy is implemented in PyTorch. The learning rate of reinforcement learning is 0.9, and the discount factor γ is set to 0.99. We train our policy on an NVIDIA GeForce RTX3080 for approximately 27,700 episodes.

We assume that after the robot takes an action at time t, it can always reach the target position at the next time t + 1, so the robot’s position update is set to:

(13) $$\begin{array}{l} p_{x}[t+1]=p_{x}[t]+v_{x}[t]\Delta t\\ p_{y}[t+1]=p_{y}[t]+v_{y}[t]\Delta t \end{array},$$

### 4.2. Simulation Setup

Our simulation environment is similar to [14,17], where the robot navigates a scene with a radius of 6 m. Since there are too few dynamic humans in [14] and the static human group in [17] is indistinguishable from static obstacles, their experimental results cannot reflect the performance of the algorithm in complex environments. In order to verify the performance of the algorithm in more complex environments, we increase the difficulty of the experiment. We added more dynamic humans in the environments. The simulated humans in the environment are controlled by ORCA, and the speed and radius of humans are random. The initial positions of all humans are randomly distributed on the circle, and their target position is on the other side of the circle. However, humans occasionally change their goals. In addition, we also added random perturbations to the start position and target position. Finally, when humans reach their goal, they do not remain stationary, but immediately proceed to a new target. These processes are to simulate the environment closer to the real environment.

We chose ORCA [5], SARL [14] and DSRNN [17] as the baseline methods, and our method is called LM-SRNN. When training was performed for SARL and DSRNN, we only modified the simulation environment, and other parameters are the same as in the original paper. In order to eliminate the performance gain brought by other factors, we remove the Human–Human interaction module in our model and implement an ablation model called ST-RNN. 

To comprehensively evaluate our model, we set up three sets of simulation experiments: the first set is an experiment in which the robot is invisible, that is, the simulated human only responds to other humans and does not respond to the robot, and there are 10 dynamic humans in the environment. The second set is an experiment where the robot is visible, in which the robot and humans interact with each other, closer to reality. As in the first set of experiments, there are 10 dynamic humans in the environment. The third set is a high-density environment. There are 20 humans in the environment, including both dynamic humans and static humans. The ratio of dynamic humans to static humans is randomly determined, and all humans will not respond to the robot. In all three sets of experiments, each method is tested using 500 random examples.

### 4.3. Quantitative Comparison

#### 4.3.1. Robot Invisibility

Navigation in an invisible environment is difficult because the robot needs to predict human behavioral trajectories in order to plan a safe path. In order to comprehensively evaluate the performance of the model, the test indicators include not only navigation success rate, navigation collision rate, and navigation time, but also discomfort frequency and minimum danger distance. Discomfort frequency refers to the percentage of the duration when the robot is too close to other humans to the total navigation time, which can reflect the frequency of the robot violating the comfort zone of other humans. The minimum distance refers to the minimum distance between the outer edge of the robot and the outer edge of other humans, which can reflect the degree of danger of the planned behavior. These two metrics can further evaluate the performance of navigation strategies in crowd navigation. Table 1 reports the experimental results in the invisible environment.

From the experimental results, it can be found that the model-based ORCA fails badly. The reason is that in an invisible environment, humans will not avoid robots, which does not meet ORCA’s reciprocity assumption. In addition, ORCAs often violate the comfort zone of other humans, and the level of danger in planning behavior is high. The reason is that ORCA only considers the current state and makes short-sighted decisions. In contrast, our LM-SRNN is trained by reinforcement learning method, and the previous series of trajectories are memorized by RNN, so the decisions made are far-sighted.

Compared with learning-based SARL, our method has a significantly higher success rate and plans a less dangerous behavior. The reason is that although SARL considers the interaction among agents, it is initialized through ORCA, which inherits the shortcomings of ORCA, and its value network is not enough to provide a good state value estimate. In contrast, our LM-SRNN is trained from scratch and does not converge to a locally optimal policy. Furthermore, our model derives Robot–Human interactions through spatio-temporal graphs and models Human–Human interactions through local coarse-grained maps, which is more efficient than simple joint modeling in SARL.

Compared with DSRNN, our method has a higher success rate, significantly shorter navigation time, and significantly lower discomfort frequency. The reason is that our method considers the impact of Human–Human interactions on robot navigation, thereby planning a more reasonable path. For the difference in path planning, we will conduct a qualitative analysis in Section 4.4.

#### 4.3.2. Robot Visible

To further validate the performance of our model, we compared it with the baseline approach in the robot visible setting. This is because the robot not only needs to understand human behavior, but also to plan a reasonable trajectory when interacting with humans. Table 2 reports the experimental results in the visible environment.

Unlike the invisible environment, the navigation success rate is greatly improved when ORCA is employed for navigation in the visible environment, which is due to the fact that this environment conforms to the reciprocity assumption of ORCA. However, since ORCA plans trajectories that are short-sighted and conservative, it still performs significantly worse than the reinforcement learning-based approach.

The performance of the reinforcement learning-based methods has been improved in the visible environment. The reason is that humans will also avoid the robot in the visible environment, and the difficulty of navigation is reduced. As with the invisible environment, our LM-SRNN still outperforms the other baseline methods.

#### 4.3.3. High-Density Environment

To further verify the performance of our model in high-density crowd environments, we increase the number of humans from 10 to 20. Since there is a timeout in navigation in such a complex environment, we introduce the indicator “Timeout”, which indicates the timeout rate of robot navigation. Table 3 shows the experimental results in a high-density environment.

As shown in Table 3, ORCA and SARL have higher timeout rates and longer navigation times in high-density environments. The reason is that the strategies adopted by ORCA and SARL initialized through ORCA are too conservative, and they will often fall into the state of robot freezing, resulting in long navigation time. Same as the robot-invisible environment and robot-visible environment, our LM-SRNN still outperforms DSRNN.

#### 4.3.4. Model Effectiveness Analysis

Furthermore, we demonstrate the effectiveness of our LM-SRNN by comparing with the ablation model ST-RNN. As shown in Table 1, Table 2 and Table 3, compared with ST-RNN, our model shows higher success rate and shorter navigation time in all environments. This is because our LM-SRNN not only considers the influence of each person’s behavior on the robot’s decision-making, but also further considers the influence of each person’s behavior by other people, so as to plan a more reasonable path.

### 4.4. Qualitative Comparison

**Figure 4:**
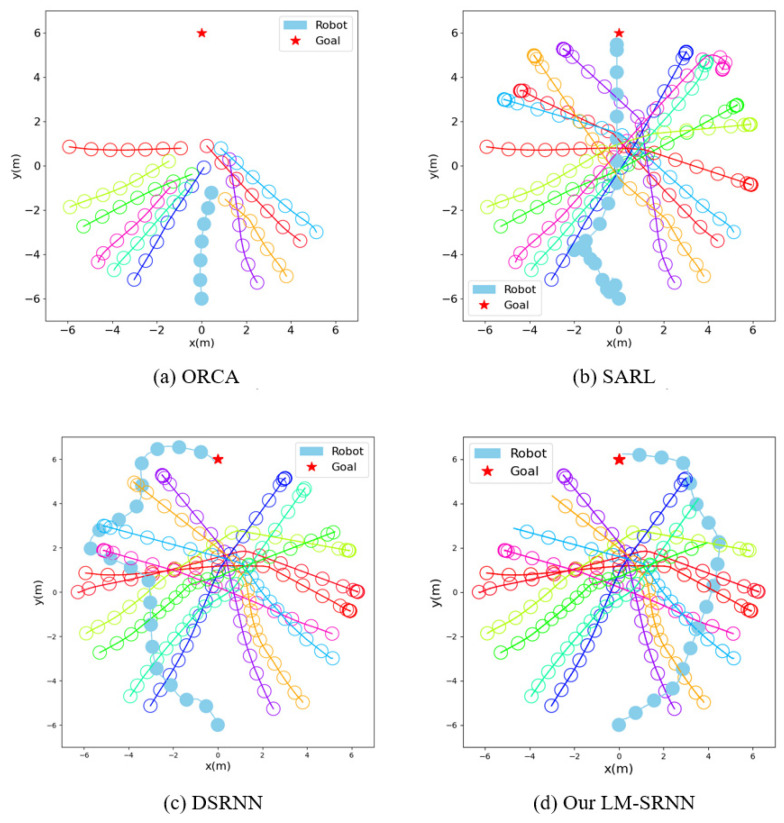
Comparison of trajectories in invisible environments. The blue solid circles in the figure represent robots, and the other hollow circles represent humans.

To further study the performance of the model, we conduct a qualitative analysis. We compare the navigation paths of different methods in the invisible environment, as shown in Figure 4. For the convenience of observation, we visualize the radius of all agents as 0.3 m. ORCA will choose the shortest path, directly into the central congested area, and eventually collide with humans. Although SARL can avoid other humans in time and successfully reach the target, it inherits the shortcomings of ORCA and will also enter the central congested area. This can lead to planning dangerous behaviors that easily violate human comfort zones. Unlike SARL, DSRNN is able to avoid centrally congested areas, but when it encounters humans, it swerves violently to pass them. Due to the inappropriate timing of avoidance and the unreasonable path chosen, the final navigation time of DSRNN is very long. In contrast, our LM-SRNN is not only able to avoid centrally congested regions, but also plans a smooth intelligent path.

**Figure 5:**
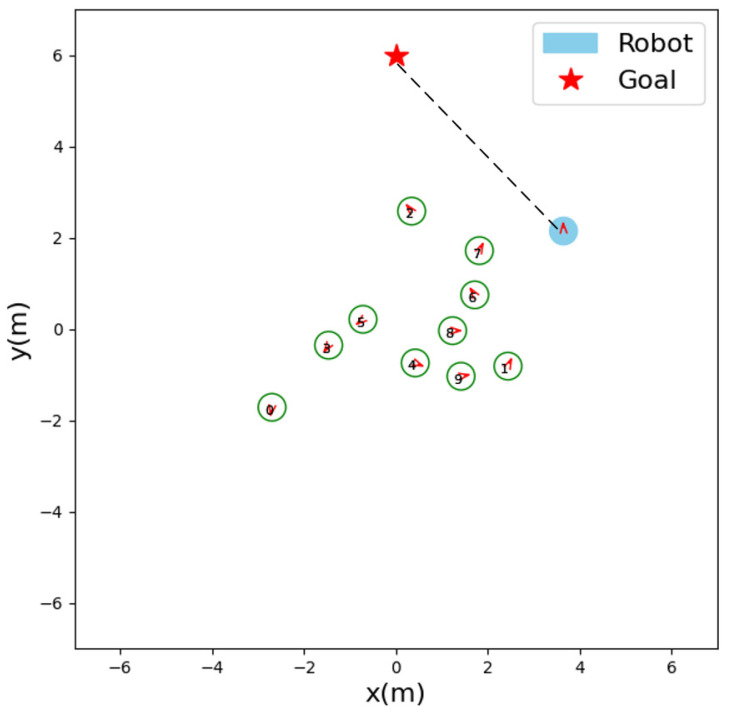
Screenshot of a moment in the navigation process. The red arrows indicate the direction of motion of the robot or human.

To take a closer look at our strategy, we analyzed how the robot was making a decision at a certain moment. As shown in Figure 5, due to the influence of human #2 and human #6, human #7 will suddenly move to the upper right. In this case, our LM-SRNN does not continue to move towards the target (the black dotted line in the figure). Instead, it moves upwards, so it does not violate the comfort zone of the human #7 and complies with social etiquette navigation norms.

## 5. Conclusions

In this work, we propose a novel LM-SRNN neural network that combines spatio-temporal maps and crowd local interaction maps in robot navigation. We build Robot–Human interactions through spatio-temporal graphs, and Human–Human interactions through local coarse-grained maps. Experimental results show that our method performs better in terms of navigation success rate and time efficiency in denser environments than the baseline method.

## Figures and Tables

**Table 1:** Experimental results in an environment where the robot is invisible. “Success” is the success rate of robot navigation. “Collision” is the collision rate for robot navigation. “Time” is the time the robot navigates. “Discomfort” is the discomfort frequency (the percentage of the duration when the robot is too close to other humans to the total navigation time). “Min Distance” is the minimum danger distance between the robot and other humans. Bold indicates the best performing model on that metric.

Method	Success	Collision	Time	Discomfort	Min Distance
ORCA [5]	0.34	0.66	15.29	0.25	0.08
SARL [14]	0.89	0.11	16.65	0.05	0.14
DSRNN [17]	0.96	0.04	18.75	0.06	0.19
ST-RNN	0.95	0.05	18.95	0.06	0.18
LM-SRNN (Ours)	**0.99**	**0.01**	16.43	**0.02**	**0.20**

**Table 2:** Experimental results in the visible environment of the robot. Bold indicates the best performing model on that metric.

Method	Success	Collision	Time	Discomfort	Min Distance
ORCA [5]	0.87	0.13	14.32	0.26	0.07
SARL [14]	0.98	0.02	14.31	0.02	0.15
DSRNN [17]	0.99	0.01	12.01	0.01	0.21
ST-RNN (Ours)	0.99	0.01	12.87	0.02	0.19
LM-SRNN (Ours)	**1.00**	**0.00**	**11.97**	**0.00**	**0.23**

**Table 3:** Experimental results in a high-density environment. “Timeout” is the timeout rate for robot navigation. Bold indicates the best performing model on that metric.

Method	Success	Collision	Timeout	Time	Min Distance
ORCA [5]	0.45	0.01	0.54	19.66	0.05
SARL [14]	0.35	0.05	0.60	29.24	0.12
DSRNN [17]	0.94	0.02	0.04	17.71	0.18
ST-RNN (Ours)	0.93	0.03	0.04	18.01	0.19
LM-SRNN (Ours)	**0.98**	**0.01**	**0.01**	**16.65**	**0.20**

## Data Availability

Not applicable.
